# Monitoring for Response to Antineoplastic Drugs: The Potential of a Metabolomic Approach

**DOI:** 10.3390/metabo7040060

**Published:** 2017-11-16

**Authors:** Jodi Rattner, Oliver F. Bathe

**Affiliations:** 1 Arnie Charbonneau Cancer Institute, University of Calgary, Calgary, AB T2N 4N2, Canada; jodi.rattner1@ucalgary.ca; 2 Department of Surgery, Tom Baker Cancer Center, University of Calgary, 1331 29th St NW, Calgary, AB T2N 4N2, Canada; 3 Department of Oncology, Tom Baker Cancer Center, University of Calgary, 1331 29th St NW, Calgary, AB T2N 4N2, Canada

**Keywords:** chemotherapy, metabolomics, response, therapeutic benefit

## Abstract

For most cancers, chemotherapeutic options are rapidly expanding, providing the oncologist with substantial choices. Therefore, there is a growing need to select the best systemic therapy, for any individual, that effectively halts tumor progression with minimal toxicity. Having the capability to predict benefit and to anticipate toxicity would be ideal, but remains elusive at this time. An alternative approach is an adaptive approach that involves close observation for treatment response and emergence of resistance. Currently, response to systemic therapy is estimated using radiographic tests. Unfortunately, radiographic estimates of response are imperfect and radiographic signs of response can be delayed. This is particularly problematic for targeted agents, as tumor shrinkage is often not apparent with these drugs. As a result, patients are exposed to prolonged courses of toxic drugs that may ultimately be found to be ineffective. A biomarker-based adaptive strategy that involves the serial analysis of the metabolome is attractive. The metabolome changes rapidly with changes in physiology. Changes in the circulating metabolome associated with various antineoplastic agents have been described, but further work will be required to understand what changes signify clinical benefit. We present an investigative approach for the discovery and validation of metabolomic response biomarkers, which consists of serial analysis of the metabolome and linkage of changes in the metabolome to measurable therapeutic benefit. Potential pitfalls in the development of metabolomic biomarkers of response and loss of response are reviewed.

## 1. Introduction

The field of oncology is currently undergoing a revolution. New antineoplastic drugs, mostly targeted drugs, are being introduced at an ever-increasing rate. As of June 2017, 361 antineoplastic drugs were in clinical trials in the United States [1]. Most of these new drugs are expected to benefit only a small fraction of patients with tumors expressing the targeted molecular features. While some of these drugs will be developed with a companion biomarker that indicates the presence of the therapeutic target, it is likely that the appearance of validated predictive biomarkers will lag. Therefore, oncologists are challenged with making a choice between a number of cytotoxic agents and an increasing number of newer agents. For any patient, that choice is mostly based on an educated guess and sound clinical judgement.

The quintessential means to personalize chemotherapy is with a predictive biomarker. Currently, there are few predictive biomarkers commonly used to make decisions, such as estrogen receptor and KRAS mutation status. There are several barriers to the development of new predictive biomarkers. First, even though the molecular target is known, its presence is not sufficient to comprise a predictive biomarker. Second, validation of any single predictive biomarker requires years, and different biomarkers are required for each drug. Third, even if a biomarker is valid, the assay for the biomarker must also perform well. This problem is illustrated by the attempts to validate ERCC1 as a predictive biomarker for chemotherapy for non-small cell lung cancer, which failed because the commercial antibody used for ERCC1 did not perform as expected [2,3]. Fourth, as different predictive biomarkers using a variety of assay techniques are introduced to practice, it is becoming difficult for any single lab to remain competent in each assay. Finally, and most importantly, predictive biomarkers identify subgroups that will not benefit from any given therapy; they do not guarantee benefit.

As a result of the inability to accurately predict who will benefit from any treatment, oncologists take an adaptive approach. A drug (or combination of drugs) is selected based on best evidence, and drug doses are selected, often based on maximum tolerated dose. Dose intensity is modified as toxicities appear. Such adverse effects can be life-threatening or debilitating; they may significantly impact quality of life; and they tend to be cumulative. Only 2–3 months after beginning a chemotherapy treatment does the oncologist evaluate treatment efficacy, and this is done with serial radiographic tests. Meanwhile, significant toxicities may have appeared and significant treatment-related costs may have incurred in the absence of any knowledge of whether the patient is benefiting.

In this review, we discuss the need for a more immediate signal of drug efficacy, and how this would enhance this adaptive approach in the treatment of solid tumors. The advantages of a biomarker-based adaptive approach are described, and the potential value of a metabolomic platform are explored. Finally, we describe some of the challenges in developing this approach, as well as ways that we can overcome those hurdles.

## 2. Serial Scans: The Standard Approach to Response Estimation

Currently, response to chemotherapy is assessed through radiographic imaging, typically computed tomography (CT) scans and magnetic resonance imaging (MRI) scans. Effective cytotoxic drugs are accompanied by a reduction in tumor size; RECIST criteria enable categorization of responses to complete response, partial response, stable disease or progressive disease [4]. In some circumstances, tumor dimensions are difficult to measure, and RECIST criteria are not conclusive. Examples include peritoneal and pleural disease, extrahepatic biliary tumors, and tumors that extend along the length of an organ such as linitus plastica. RECIST criteria are also not conclusive in patients who are treated with molecularly targeted agents such as angiogenesis inhibitors and tyrosine kinase inhibitors. Molecularly targeted agents are typically cytostatic and clinical benefit does not always accompany a reduction in tumor size. Rather, what is typically seen is a reduction in tumor density, seen on CT scan as decreased tumor attenuation. This has led to the use of Choi criteria as a means to categorize treatment response [5,6].

While CT scans and MRI scans represent the standard for following treatment response, in addition to the limitations described above, there is often a delay in the appearance of a response. As a result, it is frequent for oncologists to wait 2–3 months after starting a drug before assessing for response. By that time, a number of doses of expensive drug have been administered and significant toxicities have emerged, resulting in opportunity costs to the patient who is on ineffective therapy.

Positron emission tomography (PET) scans have been developed as an improved way of using imaging to follow response. [^18^F]-Fluorodeoxyglucose (FDG)–PET has been used effectively for monitoring response to cytotoxic therapies as well as in targeted therapies, and response can be categorized as soon as four weeks after treatment [7]. 3′-Deoxy-3′-[^18^F]-fluorothymidine–PET (FLT–PET) is an emerging technique. FLT is taken up by proliferating cells, and changes in FLT avidity have been reported as early as a week after chemotherapy [8,9]. [^18^F]-Fluorocholine–PET (FCH–PET) is based on increased choline uptake by rapidly dividing cancer cells, and may also provide an earlier signal of treatment efficacy [10,11]. While these approaches will represent improvements over CT and MRI, PET scans are not widely available. Moreover, in general, serial radiological tests are expensive; they are dependent on available infrastructure and clinical expertise; and they are inconvenient to the patient.

## 3. “Response” as a Reflection of Therapeutic Benefit

If response is to be used as an endpoint for an adaptive treatment approach, then it is essential to understand the implications of a response or a lack of response to the patient’s wellbeing. In other words, if there is a measurable response, is it likely that the patient will live longer (and with a better quality of life)? Conversely, if there is no response, does that imply that the drug is not beneficial?

Whether a response to drug confers a survival advantage is not clear, and is probably context-specific. Some studies have shown that objective response is associated with a survival benefit [12,13,14,15]. Moreover, recently, studies have demonstrated that a more immediate and more pronounced response is prognostic [16,17,18,19]. Progression-free survival (PFS) is considered a good surrogate endpoint for overall survival (OS) for some cancers [20,21,22,23,24], but not all situations [25,26]. The uncoupling of PFS and OS could be due to a number of factors, including treatment cross-over in clinical trials. It may also occur in heavily pretreated patients whose condition is already compromised. In all, the association of treatment response and survival is inconsistent.

What is consistent is the relationship between poor survival outcomes and an absence of response (or, worse, progression on treatment). This is where a test that provides the most immediate signal that a drug is ineffective would play an important role, as such a test would identify patients who are not benefiting from a drug or drug regimen, before major costs and toxicities accrue.

## 4. Biomarker-Based Methods of Response

Response biomarkers (based on blood or urine tests) represent a cheaper and more convenient alternative to radiographic methods of assessing response. (In Canada, CT costs about $1100 and MRI costs $1600 per body region; PET scan costs $3400.) If they are also more reliable, then their application to oncology practice will supplant serial scans. Moreover, if a response biomarker is an early indicator of benefit (or resistance), then the oncologist can decide relatively soon whether to persist with any drug or drug combination, before toxicities appear, before clinical deterioration occurs, and before significant monetary cost has been realized.

Protein and carbohydrate tumor markers (e.g., CEA, CA19-9, CA125, alphafetoprotein, chromogranin A) have been used to monitor the effects of systemic therapy for specific tumor types [27,28,29,30,31]. The problem with protein biomarkers is that their abundance does not necessarily reflect alterations in cellular physiology. Moreover, while they may increase quickly in response to a dramatic change in physiology, their disappearance may take much longer, limiting their usefulness as a response biomarker. Therefore, because of the difficulty in interpreting changes in protein biomarkers (including tumor markers), they have not found general use in oncology [28]. One exception is PSA, which is useful for monitoring treatment effects for prostate cancer, although it has limited effectiveness in bone disease and when cytostatic agents are administered [32].

Recently, considerable effort has been made to develop response biomarkers based on the disappearance of circulating tumor cells [33,34] and reductions in circulating nucleic acids [35,36]. These represent attractive strategies for monitoring for response, but there are some limitations. Both of these strategies rely on the presence of high pre-treatment levels of circulating tumor cells and nucleic acids, as measurable reductions from treatment are difficult to detect when baseline levels are low. Circulating tumor cells and nucleic acids are also tumor-specific. Finally, it is unclear how these approaches would perform in patients treated with cytostatic targeted therapies. A better response biomarker would respond quickly after treatment initiation, would not require a high pre-treatment baseline level, and would be more generalizable to different tumor types and different drugs.

## 5. Serial Monitoring of the Metabolome

Reprogrammed metabolism is a known hallmark of cancer [37]. In general, this metabolic reprogramming supports the high metabolic requirements of cell growth and proliferation characteristic of cancer, which require high amounts of adenosine triphosphate (ATP) and substrates for anabolic metabolism. Perhaps the best-known feature consists of enhanced glycolysis; pyruvate derived from accelerated glycolysis undergoes lactate fermentation rather than oxidative phosphorylation, even in aerobic conditions. Glutamine is a preferential fuel for tumor cells, which has a number of metabolic consequences, including an increase in the glutamate-to-glutamine ratio [38]. Fatty acid synthesis and nucleic acid synthesis are accelerated to support cell proliferation [39]. It is conceivable that effective chemotherapy would inhibit or even reverse some of these metabolic perturbations.

Taking this idea further, monitoring changes in the metabolome following initiation of chemotherapy may represent a strategy to detect changes indicative of effective treatment. Indeed, a response to chemotherapy is known to have metabolic consequences. For example, systemic therapy can result in diminished FDG uptake in tumors, reflecting a reduction in glycolysis [5,27,40,41,42,43]. Lactate dehydrogenase (which catalyzes the interconversion of lactate and pyruvate) is released from dying cells, which forms the basis of a common cytotoxicity assay used in the lab [44]. Since the metabolome changes rapidly with pathophysiological perturbations, serial analysis of the metabolome represents an attractive strategy for following treatment response.

There are additional characteristics that make a metabolomic biomarker appealing. A metabolomic biomarker is not just a string of changes in individual metabolites. Rather, it is comprised of groups of co-related metabolites that change in concert; it is a meta-biomarker. For example, changes in circulating metabolites associated with CRC might reflect alterations in metabolism that are contained within the tumor as well as alterations in the general health of the host, producing an overall “tumor signal” that reflects the extent of disease as well as its biology. In a person receiving chemotherapy, several discreet processes can be followed at once, including appearance of cell death, reduction in cell proliferation, and reduction in “tumor signal”. Importantly, because a metabolomic biomarker is a meta-biomarker, a random change in a single metabolite will not provide a false signal. A metabolomic biomarker therefore represents a powerful means of monitoring changes in an individual’s condition over time.

### 5.1. Putative Causes of Treatment-Related Changes in the Metabolome

**Figure 1:**
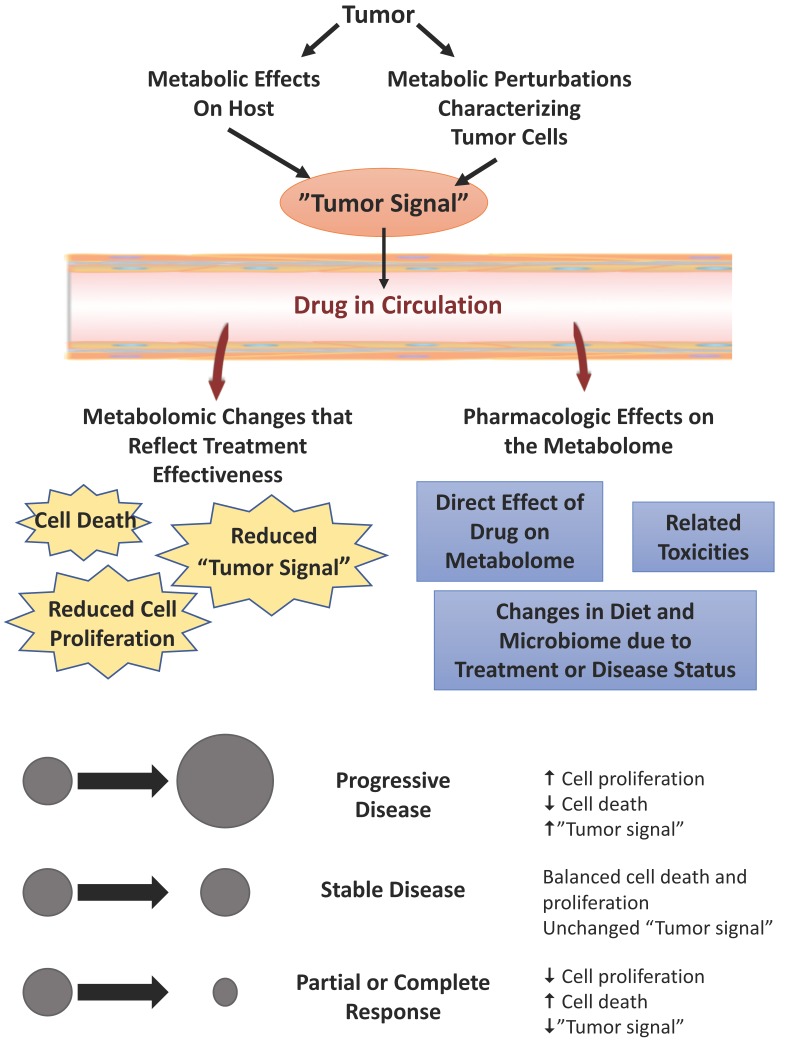
Causes of treatment-related changes in the circulating metabolome in individuals who have received antineoplastic agents. Pharmacologic effects are independent of therapeutic benefit. Antineoplastic effects are associated with benefit. Such antineoplastic effects include changes in the metabolome that are secondary to cell death, reduced cell proliferation, or a loss of “tumor signal” (which is a product of the effects on the metabolome by the tumor and by the host response to the tumor). The experimental design for discovery of a response biomarker (which signifies therapeutic benefit) will require effective linkage of metabolomic changes with objective effects on tumor progression. If treatment-related changes in the metabolome (from pre-treatment baseline) are linked to objective measures of response, then the non-specific pharmacologic effects of the drug will be effectively excluded, yielding a response biomarker.

One of the challenges of monitoring serial changes in the metabolome will be to understand the underlying cause(s) of any fluctuations. Once an antineoplastic drug is administered, a number of processes could contribute to changes in the metabolome. If blood or urine are used as the biological matrix, then it will be important to appreciate that any treatment-related alterations could originate from the tumor, the host, as well as from toxicities and idiosyncratic drug reactions (Figure 1). The challenge in developing a biomarker that signals whether the drug is beneficial is mostly related to identifying changes that do not reflect benefit, and excluding those changes that do not reflect therapeutic response.

Perhaps the most obvious causes for any change in the metabolome are the pharmacological effects. These would largely be a function of the mechanism of action, but could also be affected by dose, pharmacogenetic factors, comorbidities and concomitant drug use. If the pharmacologic effects of a drug on the metabolome are known, then the appearance of those changes would signify that the drug is being administered at a dose that effectively causes the targeted drug effects. Importantly, however, the appearance of those changes in the metabolome do not indicate that the drug is providing benefit. For that, changes associated with functional changes in tumor cells (antineoplastic effects) must be delineated.

Antineoplastic effects could comprise cell death (necrosis or apoptosis) or slowed tumor cell proliferation. Molecularly targeted agents would be expected to induce less cell death than older cytotoxic chemotherapies. Therefore, the relative degree of cell death and antiproliferative effects is dependent on the class of drugs. It follows then that, in monitoring the metabolome, the patterns of change that accompany cell death and reduced proliferation will co-exist to variable degrees.

Another potential antineoplastic effect is a loss of “tumor signal”. It is well established that patients with cancer have different metabolomes than disease-free controls. This metabolomic pattern (which we refer to as the “tumor signal”) is the product of tumor metabolism and the host response to tumor, as well as perhaps any metabolomic manifestations of factors that predispose the host to cancer. It is conceivable that effective treatment will cause a loss or a reduction in the metabolomic effects of the tumor, due to diminution of the tumor burden or perhaps due to inhibition of the metabolic perturbations that characterize cancer.

In developing a response biomarker (a biomarker that signifies benefit from chemotherapy), it will be essential to discriminate pharmacologic effects from antineoplastic effects. This can be addressed with proper experimental designs. Essential to the experimental design is the linkage of changes in the metabolome with the desired therapeutic effect, which in this case is objective evidence of tumor shrinkage or a reduction in the rate of tumor progression. Early studies should focus on relatively homogeneous patient cohorts with a single tumor type treated on a clinical trial protocol. Analytical batches must be designed to have balanced representation of samples from patients with different response categories, as well as other comparators.

It is likely that response-related changes in the metabolome are specific to tumor type and drug. It is also possible that there are several patterns of metabolomic change that appear simultaneously (depending on the existence of effects as described above). In addition, with iterative testing of samples from a large variety of conditions, it may be possible to identify some common changes in the metabolome that signal effective chemotherapy.

### 5.2. In-Vitro Studies

In-vitro studies are perhaps the easiest way to initially screen for drug-induced changes in the metabolome, as they are an ideal means to investigate the effects of a large variety of controlled conditions such as time, cell line, drug, dose, and drug mechanism of action. Integral to any cell line experiment is the functional endpoint to which drug-induced metabolomic changes are correlated. Functional endpoints relevant to a response biomarker would include cell death or a reduction in cell proliferation. Unfortunately, most in-vitro studies on drug-induced changes in the metabolome primarily involve a description of changes in the metabolome that result from the drug itself, or it is unclear whether the changes are pharmacologic or due to impairment of tumor cell expansion [45,46,47,48,49,50,51,52].

There are data on the metabolomic consequences of effective chemotherapy from a variety of cell lines treated with diverse drugs and analyzed using different analytical platforms. Using LC–MS, a fall in intracellular ATP levels and a depletion of NAD+ was observed in association with cell death from melittin and cisplatin in ovarian cancer cells [51]. Lodi et al., using ^1^H-NMR spectroscopy, characterized the metabolic fingerprints of two prostate cancer cell lines and a breast cancer cell line secondary to the pharmacological changes from a PI3K inhibitor and an HSP90 inhibitor, then identified changes that occurred due to loss of cell viability [53]. They described drug-specific changes, but there were also some changes that appeared in cells treated by both drugs, including decreased alanine, lactate and fumarate, as well as accumulation of phosphocholine and branched-chain amino acids. In human embryonic kidney cells and HepG2 hepatocellular carcinoma cells, a targeted analysis of 42 metabolites, using a direct-injection tandem mass spectrometry-based neonatal screening assay, revealed that cell death secondary to staurosporine, 5-fluorouracil and etoposide caused an array of changes [54]. Increases in alanine and glutamate appeared after treatment of all three drugs.

Most of the above cited studies evaluated changes in intracellular metabolites. Tiziani and coworkers described an NMR-based method to screen drugs for efficacy by evaluating cellular response to treatment [55]. Both intracellular and extracellular metabolites were analyzed together, which comprised a novel metabolomic response screen for kinase inhibitors. However, if a response biomarker applicable to the evaluation of clinical samples is to be derived, then it is necessary to understand effects on the extracellular compartment, for those are the metabolites most likely to be shed into the circulation. One report of metabolomic changes that accompany adriamycin treatment using GC–MS demonstrated that, in sensitive breast cancer cells, there was a distinct decline in the lactic acid levels in culture media that did not occur in resistant cells [56]. Otherwise, there is a relative paucity of in-vitro data on the metabolomic changes in the extracellular compartment that accompany effective chemotherapy.

### 5.3. In-Vivo Studies

While in-vitro studies represent a good start to understanding the metabolomic changes associated with effective chemotherapy, ultimately, in-vivo studies are essential. In-vivo studies may not recapitulate in-vitro observations for a number of reasons. There are differences in the matrices (culture media vs body fluid). Timing of analysis may have an effect. Typically, cell culture studies involve drug exposures lasting 24 h–72 h. Clinical samples may be collected at different times. In-vitro experiments occur in defined spaces, where it is possible to see changes in metabolite concentrations in the extracellular space due to fluxes with the intracellular compartment, alterations in cellular metabolism, or due to a massive release from cell lysis. In vivo, the metabolome could be affected by a variety of tumor and host factors, which is why we and others have observed that not all cancer-associated features in the circulating metabolome can be attributed to the metabolic perturbations characteristic of a cancer cell [52,57,58]. Indeed, it is well known that in-vivo observations do not recapitulate what is observed in cell line experiments [59].

A number of investigators have reported on in-vivo metabolomic changes within two weeks of starting antineoplastic drugs, but for most studies, the link between these changes and treatment effectiveness was not clearly established [60,61,62]. Metabolomic changes related to treatment effectiveness have been reported in mice and humans, using several analytical platforms [63,64,65,66].

^1^H-NMR spectroscopy was used to test murine B16 melanomas and 3LL lung cancers treated with chloroethylnitrosourea [63]. After 2–3 weeks, tumors were removed during the growth-inhibition phase and compared to untreated tumors. During growth inhibition, there was an accumulation of glucose, glutamine and aspartate (which reflects reduced cellular consumption and perhaps also the downregulation of nucleoside synthesis). Growth inhibition was also accompanied by increases in alanine, decreases in succinate and accumulation of serine-derived metabolites (glycine, phosphethanolamine, formate). This may reflect changes in the capability of the cell to catabolize alanine and serine, ordinarily used by the cancer cell to support synthesis of lipids, proteins and nucleic acids [67]. In another murine model of lung cancer reported by Weaver et al., an integrative analysis of changes in the metabolome and transcriptome was reported, including drug-specific and response-specific changes [64]. Finally, in patients with locally advanced breast cancer, tumor biopsies were evaluated by high-resolution magic-angle spinning magnetic resonance spectroscopy before and during treatment, to categorize intratumoral metabolic response [65]. Metabolic responses were related to outcome. In patients surviving ≥5 years, there was an increase in glucose, and a decrease in glycine as well as choline-containing compounds, whereas patients with poor survivals had increased tumor levels of lactate. High glucose in long-term survivors may reflect a reduction in glycolysis, and high lactate in patients with a poor prognosis may reflect continued growth and proliferation of malignant cells despite treatment.

In two instances, the metabolomic changes associated with effective chemotherapy reflected a diminished “tumor signal”. In the animal study by Weaver et al., tumor-associated changes in the lung and blood were reversed with treatment [64]. In another study involving patients with prostate cancer, LC–MS was used to identify metabolomic changes in blood that accompanied response to endocrine therapy [66]. They reported on seven metabolites that distinguished patients with prostate cancer from healthy controls (deoxycholic acid, glycogebideixycholate, tryptophan, docosapentaenoic acid, arachidonic acid, deoxycytidine triphosphate and pyridinoline). These metabolites belong to the cholesterol pathway (and are precursors for steroid hormones including androgens), as well as inflammation pathways. These same metabolites that characterized prostate cancer normalized during endocrine therapy in good responders (defined as individuals who did not develop castration-resistant prostate cancer within two years).

One analytical technique that is potentially quite interesting is matrix-assisted laser desorption/ionization (MALDI)–imaging mass spectrometry (IMS). MALDI–IMS allows the simultaneous label-free detection of multiple molecules while maintaining spatial distribution in tissues. This has a number of intriguing applications in translational research. For example, using this approach it is possible to delineate drug distribution throughout the tumor [68]. This would be particularly useful if regional patterns of cell death or growth inhibition could be mapped out using such an approach.

## 6. Early Detection of Chemoresistance

For most solid tumors, it is unusual, even after complete disappearance of the tumor, for chemotherapy to effect a cure. The more common outcome is for tumors to eventually progress as resistant cells emerge. There are many mechanisms responsible for this, but ultimately resistance emerges because of survival and growth of a clone expressing the resistance mechanism. The universal outcome is slowed rate of tumor death and outgrowth of newly vital cells. Clinically, this does not become apparent until measurable tumor expansion appears on cross-sectional imaging. At that point, the patient’s medical condition may have significantly deteriorated, which greatly affects his/her capability to receive other lines of chemotherapy. A biomarker that signals the emergence of resistance before gross tumor growth appears would therefore be advantageous.

In the context of a response biomarker, it would seem intuitive that response-related changes in the metabolome disappear and maybe even reverse as chemotherapy resistance ensues. In our own unpublished experiments, we have observed that some of the changes in metabolites associated with therapeutic benefit are extinguished or reversed when tumor growth resumes. Other mechanisms may also be responsible for resistance-related changes in the metabolome. In breast cancer cells treated with adriamycin, there were two related observations that were interesting [56]. First, when adriamycin-sensitive cells were exposed to the drug, their metabolomic profile began to take on a profile more similar to resistant cells. This suggested that emergence of resistance was accompanied by some sort of metabolic reprogramming. Second, intracellular citric acid levels decreased in response to adriamycin in sensitive cells, but increased in resistant cells. The authors postulated that treatment-related increases in citric acid levels represented a biomarker of resistance. In murine models of melanoma and lung cancer, following chemotherapy, there was a resumption of glutamine utilization in comparison to glucose utilization during the growth-recovery phase [63]. Intratumoral glucose-to-glutamine ratio (which reflects the balance of glucose and glutamine utilization) was therefore particularly instructive on the state of the tumor.

The loss of response-related metabolomic changes, or the appearance of progression-related changes, may precede gross disease progression. A biomarker based on such metabolomic events may spur the oncologist to change drugs before the patient’s health deteriorates. This has three benefits: (a) inappropriate dose escalations can be avoided, and so could the attendant toxicities; (b) inappropriately prolonged treatments can be avoided, avoiding cumulative toxicities; and (c) it will be possible to rotate to a new (potentially effective) drug regimen before gross disease progression and the associated clinical deterioration occur.

## 7. Response Biomarkers as a Disruptive Influence on Oncologic Practice

For a number of reasons, a response biomarker that determines whether a drug is benefiting an individual would be truly disruptive to the field. This would be particularly the case if that same biomarker could be used for early detection of chemoresistance. The potential impacts of a response biomarker are detailed in Table 1.

The benefits to patients and to medical oncologists can be encapsulated by minimizing exposure to ineffective drugs. Unnecessary toxicities will be avoided, performance status will be preserved, and the patient’s condition will be preserved as additional lines of chemotherapy are trialled. This is quite different than current practice, where drugs are given for a long period (even in the absence of measurable response), and cumulative toxicities slowly take their toll on performance status. As a result of this progressive deterioration, successive lines of chemotherapy are more difficult to tolerate.

The socioeconomic benefits of an effective response biomarker are substantial. Foremost will be the cost savings to the payer. Many drugs cost $5000–$10,000 per month, and the costs are rising quickly to unsustainable levels. While this may initially appear unattractive to industry, there are substantial benefits to industry as well. Phase II clinical trials could be much more cost effective if a response biomarker was used as a clinical trial endpoint; endpoints would be reached sooner, less patients may be required to complete the trial, and drugs with little activity can be quickly removed from the clinical trial queue. By rapidly identifying individuals who are benefiting from a drug, subsequent phase III trials can be better designed to include only individuals who are likely to benefit. Alternatively, if there are no obvious distinguishing features of individuals who benefit from a drug (including predictive biomarkers), then an adaptive clinical trial could be designed, using the response biomarker to select the trial cohort. Another benefit to industry is that drugs that have failed in phase III trials (because of insufficient benefit to the aggregate population) can be trialed in practice. That is, these drugs can be given on a trial basis to eligible individuals; moreover, there will be greater capability to try drugs in individuals with rare tumors, where clinical trial evidence of benefit is lacking.

## 8. Challenges in the Development of a Response Biomarker

There are some challenges related to developing a response biomarker and an ancillary biomarker that signifies chemoresistance. Serial monitoring of the metabolome must make up the experimental framework for this development. Below are further considerations in the experimental design and in risk mitigation.

### 8.1. Selecting the Best Analytical Platforms for Biomarker Discovery

A number of analytical platforms have been used for metabolomics work. Details of the advantages and disadvantages of each platform is outside of the scope of this review. However, in general, it is essential that the technology can be adapted to a clinical laboratory, if the ultimate goal is to create a clinical test. Therefore, it must be easy to use, there must be high-throughput capability, and the spatial footprint must not be excessive. While, at first glance, instrumentation that has the resolution and sensitivity to detect more compounds is advantageous, measurement of more compounds comes with the cost of increased signal-to-noise, resulting in more difficulty in constructing a stable metabolomic model. In our own work, we have preferred to use gas chromatography–time of flight–mass spectrometry (GC–TOF–MS), which has sufficiently high resolution to reproducibly detect several hundred compounds and takes up the space of a desktop. Another essential feature of a clinical assay is that it is quantifiable and reproducible, which requires the use of internal controls for any of the mass spectrometry-based modalities. Finally, the cost of the final assay must be significantly less than the cost of serial radiographic scans. Currently, the main obstacle that keeps costs high is the requirement for sophisticated technical and statistical analyses related to metabolomic biomarker work. Therefore, it will be imperative to devise a workflow that enhances the capability to analyze and interpret many samples in a high-throughput fashion.

### 8.2. Distinguishing Changes in the Metabolome that Reflect Treatment Effectiveness from Changes that Are Due to Pharmacological Effects

As discussed above, in the literature, there are numerous descriptions of changes in the metabolome that accompany administration of various antineoplastic drugs. However, most experimental designs do not enable one to distinguish treatment-related changes that specifically accompany effective treatment. To do that, it will be imperative to link metabolomic changes to reductions in tumor size or density (on CT or MRI), or to reduced avidity to radioisotopes on PET scans.

### 8.3. Accounting for Genetic Differences, Dietary Variations and Environmental Influences

Genetic traits of the individual probably affect the metabolome in health and disease, as would diet and various environmental influences. Indeed, in most metabolomic biomarkers we have so far developed, gender differences have been identified. The advantage of doing serial measurements is that the intra-individual comparisons reduce the influence of genetics and environment. On the other hand, it is conceivable that treatment with toxic drugs or disease progression can each cause clinical deterioration, which may cause dietary changes or may cause the appearance of other confounding changes in the metabolome.

### 8.4. Longitudinal Assessment of the Metabolome: Analytical Challenges

While serial monitoring for changes in the metabolome within an individual minimizes the effects of genetics and diet on the metabolomic biomarker, better methods will need to be devised to monitor the multiple (co-related) metabolites over time. The challenge lies in identifying the patterns of changes that accompany therapeutic benefit and the loss of these same changes as the benefit is extinguished with the emergence of chemoresistance.

### 8.5. Response to Cytotoxic Agents vs. Cytostatic Agents

The most common chemotherapeutic agents used in clinical practice throughout the last few decades have mainly consisted of cytotoxic chemotherapy agents. These agents were developed and designed to destroy cancer cells, eradicating solid tumors. As such, tumor shrinkage has been the mainstay common end-point of determining response to these agents. More recently, there has been a rapid expansion of novel non-cytotoxic agents with various mechanisms of action that specifically target an ever-expanding array of molecules. These molecularly targeted cytostatic agents are characterized by their ability to target specific pathways that encourage tumor progression. Administration of cytostatic agents results in the inhibition of tumor growth and proliferation, without the common toxicities associated with cytotoxic agents. However, cytostatic drugs may not result in the physical shrinkage of the tumor in size. Rather, there is a change in tumor density that often accompanies effective treatment. Therefore, the measure of treatment effectiveness (response) must take into consideration the likely effects of these drugs on tumors.

### 8.6. Assessment of Stable Disease

In patients treated with either cytotoxic agents or targeted agents, the “stable disease” condition represents a potential challenge. That is, in the absence of tumor shrinkage or growth after treatment, it is difficult to delineate whether this stability is due to indolent tumor biology or stabilization of growth secondary to treatment. There is no simple way to distinguish these two possibilities. However, an additional outcome variable could be monitored, such as progression-free survival, which is generally longer in patients benefiting from treatment.

### 8.7. The Kinetics of Response

Another challenge will be to understand the kinetics of any treatment-related changes. It is conceivable that the types of metabolites that significantly change with treatment vary with time after drug is administered. There are many reasons for this. Different metabolic functions may be affected by the drug at different time intervals. Drug metabolism, tumor burden and off-target toxicities could affect the kinetics of these changes. Therefore, as metabolomic response biomarkers are developed, it will be imperative to understand the kinetics of these changes. A clinically useful biomarker would appear soon after the drug has been initiated, and would remain stable as long as the tumor does not progress. Once the kinetics of any biomarker are understood, it will be important to standardize the sample collection times after each drug dose.

### 8.8. Drug-Specific vs. Generalizable Features of Response

It is anticipated that each drug or drug combination will be associated with specific response-associated metabolomic changes due to differences in mechanisms of action. However, it is also possible that there may be some response-associated metabolomic changes that are generalizable, which would be particularly useful. For example, for cytotoxic agents, a common feature might be the metabolomic changes that are associated with cell death. For cytostatic agents, the metabolomic effects of reduced cell proliferation may represent a common feature.

## 9. Conclusions

For many cancers, the chemotherapeutic options are rapidly expanding. It has become clear that there is considerable inter-individual variability in chemosensitivity. Therefore, there is a need to individualize drug therapy. Ideally, a predictive biomarker would aid in the selection of agents. However, validated predictive biomarkers are not widely available. Therefore, an alternative approach to individualizing therapy would be an adaptive approach, which involves modifying chemotherapy according to the appearance of signs of benefit or loss of benefit. This approach is already being employed, but currently involves the use of radiographic tests to inform the oncologist. A biomarker-based approach to this adaptive strategy may have some advantages, especially if the biomarker appears early and reliably. The metabolome is very sensitive to changes in medical condition and therefore has the potential to form the basis for such a biomarker. Serial monitoring of the metabolome, looking for changes that correlate with clinical benefit, represents the most attractive strategy for the development of biomarkers of response and emergence of chemoresistance.

## Figures and Tables

**Table 1:** Potential benefits of a response biomarker for antineoplastic agents.

Beneficiary	Benefits
Benefits to the Patient	Minimize exposure to ineffective and potentially harmful chemotherapy drugs. Avoidance of unnecessary toxicities, improving quality of life and possibly survival. A response biomarker that reflects chemosensitivity may expand therapeutic options available by identifying subpopulations that will directly benefit from such drugs, expanding antineoplastic formulary for individuals. Preservation of performance status will facilitate administration of later lines of therapy.
Effects on Clinical Practice	Therapy will be individualized using a biomarker that reflects response, resistance and sensitivity to therapeutic administration. Will enable dose titration. The lowest effective dose for an individual could be administered, thus reducing treatment-related toxicities. Early detection of chemoresistance will have the following benefits: (a) inappropriate dose escalations can be avoided, and so could the attendant toxicities; (b) inappropriately prolonged treatments can be avoided, avoiding cumulative toxicities; (c) it will be possible to rotate to a new (potentially effective) drug regimen before gross disease progression and the associated clinical deterioration occur. Will enable improved monitoring of treatment effect in patients with malignant conditions that are difficult to gauge radiologically (e.g., peritoneal disease, malignant effusions).
Socioeconomic Benefits	Payers (including insurance companies, governments and patients) will pay much less for ineffective drugs. Patients whose quality of life is preserved and whose disease is controlled with less toxicity will be more likely to be able to resume normal activities, including work. Novel drug development will be less expensive and more efficient, which may translate to development of more, less-costly drugs.
Benefits to Industry	Clinical trial designs would be revolutionized: the availability of a biomarker of chemosensitivity will provide a new trial endpoint, enabling identification of appropriate doses and patient populations with less harm to trial patients in phase I trials. Opportunity for industry to reintroduce some drugs to clinical practice that have efficacy in CRC but insufficient benefit to the aggregate. Phase II trials can be performed more quickly, using the biomarker as a surrogate marker for benefit. Such trials would also be less onerous on trial participants. This would result in new drugs being screened and introduced more quickly and efficiently to the market, translating to more, less-costly drugs. There will be less need for predictive biomarkers, which take years to develop and validate.

## Abbreviations

The following abbreviations are used in this manuscript:

CT	Computed tomography
FCH	Fluorocholine
FDG	Fluorodeoxyglucose
FLT	Fluorothymidine
GC	Gas chromatography
LC	Liquid chromatography
MALDI-IMS	Matrix-assisted laser desorption/ionization imaging mass spectrometry
MS	Mass spectrometry
NMR	Nuclear magnetic resonance
OS	Overall survival
PET	Positron emission tomography
PFS	Progression free survival
